# Use of the European Organisation for Research and Treatment of Cancer multiple myeloma module (EORTC QLQ-MY20): a review of the literature 25 years after development

**DOI:** 10.1038/s41408-023-00815-9

**Published:** 2023-05-16

**Authors:** K. Forde, K. Cocks, J. R. Wells, I. McMillan, C. Kyriakou, EORTC Quality of Life Group

**Affiliations:** 1 Adelphi Values, Patient-Centered Outcomes, Bollington, Cheshire, UK; 2 Previously of Adelphi Values, Patient-Centered Outcomes, Bollington, Cheshire, UK; 3 Department of Haematology, University College London Hospitals NHS Foundation Trust, London, UK

**Keywords:** Quality of life, Myeloma

## Abstract

The European Organisation for Research and Treatment of Cancer Quality of Life Multiple Myeloma Questionnaire (EORTC QLQ-MY20) was developed in 1996 to assess health-related quality of life (HRQoL) in patients with multiple myeloma. Since its development new therapies have prolonged survival in patients with myeloma and new combination agents are likely to impact HRQoL outcomes and its measurement.

The aim of this review was to explore the use of the QLQ-MY20 and reported methodological issues.

An electronic database search was conducted (1996-June 2020) to identify clinical studies/research that used the QLQ-MY20 or assessed its psychometric properties. Data were extracted from full-text publications/conference abstracts and checked by a second rater.

The search returned 65 clinical and 9 psychometric validation studies. The QLQ-MY20 was used in interventional (*n* = 21, 32%) and observational (*n* = 44, 68%) studies and the publication of QLQ-MY20 data in clinical trials increased over time. Clinical studies commonly included relapsed patients with myeloma patients (*n* = 15, 68%) and assessed a range of combinations therapies.

QLQ-MY20 subscales (disease symptoms [DS], side effects of treatment [SE], future perspectives [FP], body image [BI]) were defined as secondary (*n* = 12, 55%) or exploratory (*n* = 7, 32%) trial endpoints, particularly DS (*n* = 16, 72%) and SE (*n* = 16, 72%). Validation articles demonstrated that all domains performed well regarding internal consistency reliability (>0.7), test-reset reliability (intraclass correlation coefficient > =0.85), internal and external convergent and discriminant validity. Four articles reported a high percentage of ceiling effects in the BI subscale; all other subscales performed well regarding floor and ceiling effects.

The EORTC QLQ-MY20 remains a widely used and psychometrically robust instrument. While no specific problems were identified from the published literature, qualitative interviews are ongoing to ensure new concepts and side effects are included that may arise from patients receiving novel treatments or from longer survival with multiple lines of treatment.

## Introduction

Multiple Myeloma (MM) is a haematological cancer that affects multiple organs and is associated with complex symptoms [1]. However, due to treatment option advances, MM survival rates have significantly improved in the past 25 years [2–4]. Despite the constantly evolving treatment landscape for MM, it remains an incurable and progressive disease, that requires either continuous or intermittent therapies to maintain disease stability and sustain or prolong the survival [5].

Disease symptoms, in addition to treatment side effects caused by multiple lines of therapies, can severely impact on patient’s wider health-related quality of life (HRQoL). For example, fatigue and pain are physical symptoms commonly reported by patients with myeloma which significantly impair HRQoL [6, 7]. In addition to extended survival, it is important to understand how new and combination treatments may affects patients’ lives, therefore, it is recognised that patient-reported outcome (PRO) measures are vital to assess in clinical trials and in the management of MM [8].

The European Organisation for Research and Treatment of Cancer Quality of Life Multiple Myeloma Questionnaire (EORTC QLQ-MY20), developed in 1999, is a MM specific PRO measure consisting of 20 items within four domains (disease symptoms [DS], side effects of treatment [SE], future perspectives [FP] and body image [BI]) [9]. The original module QLQ-MY24, released in 1996, included 4 additional items under the domain of Social Support (SS) that was subsequently removed due to observed ceiling effects [10]. The QLQ-MY20 module is used in conjunction with the EORTC Core Quality of Life Questionnaire (QLQ-C30) designed for use in oncology patients more generally. The MM module has been translated into over 70 language versions [11], is a MM-specific measure used most globally and is one of the most extensively validated instrument for use in MM clinical research [10, 12].

Since the module’s development, the treatment for MM has changed [13]. The original validation of the QLQ-MY20 was largely in newly diagnosed patients and the module was focused on the expected side effects of conventional chemotherapy and steroids when it was originally developed [9]. The conventional chemotherapy in 1999 was mainly melphalan, cyclophosphamide, vincristine and doxorubicin. Although it is recognized that patients with myeloma can be treated with a variety of different chemotherapy drugs and regimens, it was felt that the side effects of conventional chemotherapy and steroids may more adversely affect the HRQoL of the patients for a longer period of time. However, after 1999, no-chemotherapy treatments (proteasome inhibitors, immunomodulatory drugs, monoclonal antibodies and other novel agents) have been introduced. The increase in survival rates coupled with the rapid progression in therapeutic options for patients with myeloma have implications for the HRQoL outcomes and side effects for this population. Osborne et al published a review in 2012 identifying issues important to patients and whether existing instruments comprehensively cover the current treatment landscape and patient experience [12]. While the QLQ-C30 and QLQ-MY20 were acknowledged as the instruments which had good conceptual coverage and had undergone the most extensive validation in patients with myeloma, no instruments were identified as covering all issues relevant to patients, signifying the need for a MM module update that will represent HRQoL taking into account current therapy issues and HRQoL concerns to patients today.

The EORTC guidelines provide a four-phase framework for updating existing modules [14]. As part of Phase I (generation of QoL issues), a literature review assessing the use of the QLQ-MY20, and any reported methodological issues was performed. The following article details this literature review which aimed to explore:

1. In which types of clinical studies the module has been used
2. To what extent has the module been used in both newly diagnosed and relapsed patients
3. The types of treatments/therapies the module has been used to assess
4. How and where the module-related endpoint is positioned within randomised controlled trials (RCTs)
5. How the module results are reported, and the prominence given to these results
6. The statistical results from QLQ-MY20 subscales in RCTs
7. PRO limitations identified from interventional studies and validity/reliability issues raised in psychometric validation studies

## Methods

### Literature search, eligibility criteria and screening

The primary search was conducted using the Ovid SP platform, accessing the electronic bibliographic databases: Medline, EMBASE and PsycINFO. Searches combined the use Keyword search (i.e., reference is identified if it includes the specified term within its bibliographic reference) and a Subject Heading search. Subject Headings are a controlled set of terms used in bibliographic databases to index articles by topic. A supplementary search in Google scholar was also performed and references that had not been previously identified were reviewed for inclusion. Only papers published between 1996 and 2020 were sought as this reflects when the MM module (MY24) was first released. The searches sought publications referencing ‘Multiple Myeloma’ in addition to ‘MY20’, ‘MY24’ or ‘EORTC’ or containing reference to the QLQ-MY20 domains.

See supplementary materials [1] for search strings.

Abstracts were included if they were reporting a clinical study of any design that generated data using the QLQ-MY20/24 or a study to evaluate the QLQ-MY20/24, including the assessment of the psychometric properties of the module (validation study). Only abstracts reporting original research were included thus reviews, conference proceedings and book chapters were excluded. The full-text publications were sought for all references meeting these criteria. When a single study was referenced across multiple references only the most comprehensive or relevant publication (e.g., HRQoL focused) was retained. Clinical studies were categorized as interventional (i.e., RCT’s, clinical trial – single-arm, clinical trial – cross over) or observational (i.e., cross-sectional and longitudinal/cohort) study designs.

### Data extraction

General information (e.g., author, title and year and location of the study) was collected for all studies. For all clinical studies information about the disease severity (i.e., newly diagnosed/relapsed), and other clinical outcome assessments (COAs, including patient-reported outcomes) used was extracted. For trials (RCT’s, single, and cross-over arm) further information about the study design, reporting and presentation of results were extracted. Further in-depth extraction of RCTs was performed, including type of statistical analysis on QLQ-MY20 data and comparisons between groups. For validation studies data on the instrument structure and data distribution, reliability, validity and ability to detect change/interpretation of change scores was extracted.

### Interrater agreement

Data extraction was initially performed by one reviewer, the indications of the first reviewer were subsequently checked by a second reviewer. Any cases of disagreement or uncertainty were then discussed, and consensus was established in all instances by the study team based on the inclusion criteria. For the extraction of statistical data, all data extracted was checked by a statistician to ensure accuracy.

## Results

**Fig. 1:**
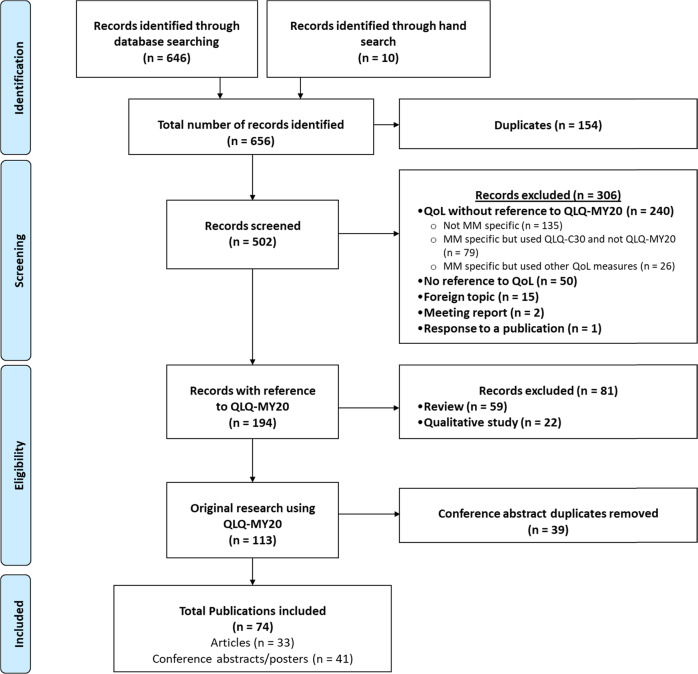
Flow diagram of the abstract screening process.

The search yield 502 unique records (Fig. 1) of which 74 publications were taken forward for review (33 full-text articles and 41 conference abstracts).

### Study designs where QLQ-MY20 is used

Table 1 provides an overview of study design where the QLQ-MY20 was used and the country in which the author team were affiliated. The studies had a wide international spread and in recent years there has been a growth in scientific publication on the use of the QLQ-MY20 in both clinical and instrument validation studies. There has been an increase in the use of the QLQ-MY20 in RCTs, single-arm clinical trials and cross-sectional observational studies over time.

**Table 1:** Study design and country.

**Study design**	***n*** = **number of studies**				
	**Total** ***N*** = **74**	**2006–2010**	**2011–2015**		**2016–2020**
**Clinical studies**	**65**	**5**	**29**		**31**
*Interventional*					
Randomised controlled trial (RCT)	15	0	5		10
Clinical trial – single arm	5	1	1		3
Clinical trial – cross over	1	0	1		0
*Observational*					
Cross-sectional	26	4	10		12
Longitudinal/cohort	18	0	12		6
**Instrument validation studies**	**9**	**1**	**5**		**3**
**Country**^**a**^	**Total** ***N*** = **74**	***n*** = **number of interventional studies**		***n*** = **number of observational studies**	
International (>2 countries)	**19**	11		8	
USA	**7**	2		5	
UK	**7**	4		3	
Netherlands	**6**	1		5	
Italy	**4**	0		4	
Denmark	**3**	2		1	
Germany	**3**	0		3	
Korea	**3**	0		3	
Spain	**2**	1		1	
Mexico	**2**	0		2	
France	**2**	0		2	
Belgium	**1**	0		1	

^a^Country is defined by the country in which the corresponding author and/or author team are affiliated. Affiliate countries where only one observational study using the QLQ-MY20 was conducted: Algeria, Australia, Austria, Canada, Ireland, Lithuania, Portugal, and Turkey

### QLQ-MY20 instrument use in observational and interventional studies

When stated, interventional and observational studies included either exclusively relapsed patients (*n* = 24/43, 55.8%, 14 of which were interventional), newly diagnosed patients (*n* = 10/43, 23.3%, seven of which were interventional), and a mix of newly diagnosed and relapsed patients (*n* = 9/43, 20.9%, none of which were interventional). Over time, both observational and clinical trials increasingly utilized the QLQ-MY20 with samples of relapsed patients and mixed samples of newly diagnosed and relapsed patients.

With the exception of two observational studies [15, 16], all 65 studies used the QLQ-MY20 in conjunction with the EORTC QLQ-C30 as required by the EORTC modular measurement approach. The COAs used in conjunction with the EORTC QLQ-C30 [17] and QLQ-MY20 [10] module largely assessed peripheral neuropathy (e.g., FACT-GOG-Ntx) [18], HRQoL (e.g., EQ-5D-5L) [19], emotional wellbeing, particularly anxiety and depression (e.g., HADS) [20], fatigue (e.g., FACIT) [21], sleep quality (e.g., PSQI) [22] and functional impairment (e.g., KPS) [23]. A complete list of COAs used in conjunction with the QLQ-MY20 can be found in the supplementary material (Supplementary Table 1).

### QLQ-MY20 instrument use in observational studies

See Supplementary Table 2 for a summary of observational studies that included the QLQ-MY20.

### QLQ-MY20 instrument use in interventional trials

Table 2 provides a summary of the characteristics of the interventional studies that used the QLQ-MY20 (*n* = 21). QLQ-MY20 subscales were most commonly defined in studies as secondary (*n* = 11/21, 52%) or exploratory (*n* = 6/21, 29%) endpoints.

**Table 2:** Summary of the *n* = 21 interventional trials (randomized controlled trial or clinical trial-single arm/cross-over) identified by the literature review.

Time categories	Author, year of publication	Title	Study design/ type	Study phase	QoL sample size	Study author(s) affiliated location	Disease stage	QLQ-MY20 Endpoint heirarchy	QLQ-MY20 subscales reported	Additional questionnaires reported	Presentation of QoL results	PRO limitations raised
	Publication type: Article											
2006–2010	Dubois D. et al. [32]	Descriptive and prognostic value of patient-reported outcomes: The bortezomib experience in relapsed and refractory multiple myeloma.	Single-trial arm	Phase II	144	Belgium	1st or subsequent relapses	Not reported	DS, SE, FP (not as endpoint)	FACT-GOG-Ntx FACIT	Text, table and figure	–
2011–2015	Verelst S. et al. [33]	Effect of thalidomide with melphalan and prednisone on health-related quality of life (HRQOL) in elderly patients with newly diagnosed multiple myeloma: A prospective analysis in a randomized trial (HOVON 49).	RCT	Phase III	284	Netherlands	Newly diagnosed	Secondary	DS, SE, FP, BI	No additional questionnaires reported	Text, table and figure	–
	Dimopoulos M.A. et al. [34]	Lenalidomide, melphalan, and prednisone, followed by lenalidomide maintenance, improves health-related quality of life in newly diagnosed multiple myeloma patients aged 65 years or older: results of a randomized phase III trial	RCT	Phase III	459	International	Newly diagnosed	Secondary	DS, SE, FP, BI	No additional questionnaires reported	Text, table and figure	–
	Alegre A. et al. [35]	Efficacy, safety and quality-of-life associated with lenalidomide plus dexamethasone for the treatment of relapsed or refractory multiple myeloma: the Spanish experience.	Single-trial arm	Phase III	63	Spain	1st or subsequent relapses	Secondary	DS, SE, FP, BI	No additional questionnaires reported	Text and figure	The single treatment arm design, and lack of comparator groups limits the strength of the implications that can be drawn from the HRQoL analysis
	Delforge M. et al. [36]	Health-related quality-of-life in patients with newly diagnosed multiple myeloma in the FIRST trial: Lenalidomide plus low-dose dexamethasone versus melphalan, prednisone, thalidomide.	RCT	Phase III	1623	International	Newly diagnosed	Secondary	DS, SE, FP, BI	EQ-5D-5L	Text, table and figure	HRQoL data was not collected beyond 18 months of treatment; therefore, conclusions cannot be drawn on the effects of long-term continuous Rd treatment on patients’ HRQoL. Further analyses should consider meaningful change at the individual patient level
	Weisel K. et al. [37]	Pomalidomide and Low-Dose Dexamethasone Improves Health-Related Quality of Life and Prolongs Time to Worsening in Relapsed/Refractory Patients with Multiple Myeloma Enrolled in the MM-003 Randomized Phase III Trial.	RCT	Phase III	433	International	1st or subsequent relapses	Secondary	DS, SE	EQ-5D-5L	Text and figure	-
2016-2020	Stewart A.K. et al. [38]	Health-related quality-of-life results from the open-label, randomized, phase III ASPIRE trial evaluating carfilzomib, lenalidomide, and dexamethasone versus lenalidomide and dexamethasone in patients with relapsed multiple myeloma.	RCT	Phase III	696	International	1st or subsequent relapses	Exploratory	DS, SE	No additional questionnaires reported	Text, table and figure	Differential dropout
	Leleu X. et al. [39]	Patient-reported health-related quality of life from the phase III TOURMALINE-MM1 study of ixazomib-lenalidomide-dexamethasone versus placebo-lenalidomide-dexamethasone in relapsed/refractory multiple myeloma.	RCT	Phase III	670	International	1st or subsequent relapses	Secondary	DS, SE, FP, BI	No additional questionnaires reported	Text and figure	The authors note that questions currently included in the HRQoL assessments may not be sensitive enough to pick up variations in HRQoL depending on the type of treatment administered
	Richardson P.G. et al. [40]	Patient-reported outcomes of multiple myeloma patients treated with panobinostat after > =2 lines of therapy based on the international phase 3, randomized, double-blind, placebo-controlled PANORAMA-1 trial.	RCT	Phase III	147	USA	1st or subsequent relapses	Secondary	DS, SE, FP, BI	FACT-GOG-Ntx	Text, table and figure	Lack of established guidelines for collecting and analysing PRO data in MM complicates comparisons with results from other studies
	Royle K.-L. et al. [41]	Quality of life during and following sequential treatment of previously untreated patients with multiple myeloma: findings of the Medical Research Council Myeloma IX randomised study.	RCT	Phase III	1819	UK	Newly diagnosed	Exploratory	DS, SE, FP, BI	No additional questionnaires reported	Text, table and figure	Alternative definitions of MID make comparison between studies very difficult and research less meaningful. A consensus on such measurements is required. High proportion of missing data a limitation
	Cella D. et al. [42]	Impact of elotuzumab treatment on pain and health-related quality of life in patients with relapsed or refractory multiple myeloma: results from the ELOQUENT-2 study.	RCT	Phase III	630	International	1st or subsequent relapses	Exploratory	DS, SE	No additional questionnaires reported	Text and table	Low levels of pain at baseline meant that demonstrating clinically meaningful improvement in pain was challenging. Dosing convenience may affect HRQOL but not adequately measured by current PRO instruments
	Ahmedzai S.H. et al. [43]	Patient-reported outcome results from the open-label, randomized phase III myeloma X trial evaluating salvage autologous stem-cell transplantation in relapsed multiple myeloma.	RCT	Phase III	171	UK	1st or subsequent relapses	Secondary	DS, SE, FP, BI	BPI-SF S-LANSS	Text, table and figure	Reliance on paper-based questionnaires and associated adherence problems
	Moreau P. et al. [44]	Convenience, satisfaction, health-related quality of life of once-weekly 70 mg/m2 vs. twice-weekly 27 mg/m2 carfilzomib (randomized A.R.R.O.W. study).	RCT	Phase III	469	International	1st or subsequent relapses	Exploratory	DS, SE, FP, BI	EQ-5D-5L	Text, table and figure	The authors noted the higher incidence of grade 3 and higher treatment-emergent adverse events in one arm did not translate into worse HRQoL
	Nielsen L.K. et al. [45]	Health-related quality of life in transplant ineligible newly diagnosed multiple myeloma patients treated with either thalidomide or lenalidomide-based regimen until progression: a prospective, open-label, multicenter, randomized, phase 3 study	RCT	Phase III	596	International	Newly diagnosed	Not reported	DS, SE, FP, BI	No additional questionnaires reported	Text, table and figure	The authors noted a discrepancy between patient reported QLQ-MY20 item ‘tingling hands and feet’ and clinical reported peripheral neuropathy events. They suggested use of the QLQ-CIPN20 may improve concordance between patients and physicians
	Publication type: Conference abstract											
2011-2015	Dimopoulos M.A. et al. [46]	Vantage 088: Vorinostat in Combination with Bortezomib in Patients with Relapsed/Refractory Multiple Myeloma: Results of a Global, Randomized Phase 3 Trial.	RCT	Phase III	Not reported	International	1st or subsequent relapses	Exploratory	–	No additional questionnaires reported	Text, table and figure	–
	Ryman N. et al. [47]	Interim analysis of a study to investigate safety, quality of life (QOL), patient satisfaction & preference with domiciliary versus day ward administration of bortezomib.	Trial - crossover	Not reported	16	UK	1st or subsequent relapses	Not reported	–	–	–	–
2016-2020	Ludwig H. et al. [48]	Health related quality of life results from the open-label, randomized, phase III endeavor trial evaluating carfilzomib and dexamethasone versus bortezomib and dexamethasone in patients with relapsed or refractory multiple myeloma.	RCT	Phase III	Not reported	International	1st or subsequent relapses	Exploratory	DS, SE	FACT-GOG-Ntx	–	Differences in grade 2 and above peripheral neuropathy AEs did not translate into patient-reported differences on the neurotoxicity subscale of the FACT-GOG-Ntx. Missing data may have contributed to the lack of clinically meaningful difference
	Ludwig H. et al. [49]	Health-related quality of life in patients with relapsed/refractory multiple myeloma during treatment with ixazomib-thalidomide-dexamethasone followed by ixazomib maintenance.	Single-trial arm	Phase II	77	International	1st or subsequent relapses	Not reported	–	No additional questionnaires reported	–	–
	Rifkin R.M. et al. [50]	Tourmaline US-MM6, an open-label, single-arm, multicenter study evaluating the effectiveness and safety of ixazomib in combination with lenalidomide and dexamethasone (iRD) in patients (pts) with newly diagnosed multiple myeloma (NDMM) switching from a bortezomib-based triplet induction regimen.	Single-trial arm	Phase IV	160	USA UK	Newly diagnosed	Secondary	–	TSQM-9	–	–
	Nielsen L.K. et al. [24]	Clarithromycin added to the VCD regimen causes reduced health-related quality of life in multiple myeloma patients.	RCT	Phase II	55	Denmark	Newly diagnosed	Secondary	DS, SE, FP, BI	FACT-GOG-Ntx	–	Poor questionnaire completion rate, differential dropout, limited number of patients with higher grade AEs reporting a global QOL score
	Eshoj H.R. et al. [51]	Health-related quality of life in multiple myeloma patients with first relapse treated with Carfilzomib-based re-induction and salvage autologous stem cell transplantation: Data from a Nordic phase II trial.	Single-trial arm	Phase II	92	Denmark	1st or subsequent relapses	Secondary	DS, SE, FP, BI	FACT-GOG-Ntx	–	–

*BI* body image, *BPI-SF* Brief Pain Inventory-Short Form, *DS* disease symptoms, *EQ-5D-5L* EuroQol-5 Dimension-5 Level, *FACIT* The Functional Assessment of Chronic Illness Therapy, *FACT-GOG-Ntx* Functional Assessment of Cancer Therapy/Gynecologic Oncology Group-Neurotoxicity, *FP* future perspectives, *HRQoL* Health-related quality of life, *IRD* Ixazomib, revlimid/lenalidomide and dexamethasone, *MM* Multiple myeloma, *MPR-R* lenalidomide Melphalan/prednisone plus lenalidomide, *MPT-T* Melphalan/prednisone plus thalidomide, *NDMM* newly diagnosed multiple myeloma, *Pts* Patients, *QoL* Quality of life, *RCT* Randomised control trial, *SE* side effects, *S-LANSS* Self-report version of the Leeds Assessment of Neuropathic Symptoms and Signs Pain Scale, *TSQM-9* Treatment Satisfaction Questionnaire for Medication-9 items), ‘-‘ denotes information not reported

For each study identified, the summary table details the type of publication, author and year of publication, article/abstract title, study design/type, study phase, quality of life sample size, study author(s) affiliated location, patients disease stage, where the QLQ-MY20 had been positioned as an endpoint, the QLQ-MY20 domains reported, additional questionnaires reported, the presentation of QoL results and PRO limitations raised. The table is presented in chronology according to the paper’s year of publication.

Trends over time (for the reporting periods 2006–2010, 2011–2015, and 2016–2020) were assessed across interventional studies and four notable trends were observed. Over time, the proportion of RCTs, relative to single-trial arm and cross-over trials, increased from *n* = 0 between 2006 and 2010 to *n* = 5/7 between 2011 and 2015 to *n* = 10/13 between 2016-2020. Similarly, the number and proportion of trials utilizing a sample of patients who have experienced their 1st or subsequent relapses, relative to being newly diagnosed, increased over time from *n* = 1/2 between 2006 and 2010 to *n* = 4/7 between 2011 and 2015, and *n* = 9/13 between 2016-2020. The average QoL sample size increased from 144 between 2006 and 2010 to 479 and 465 between 2011 and 2015 and 2016 and 2020 respectively. In recent years, there has also been more questionnaires used in conjunction with the QLQ-MY20; between 2006 and 2010 only two additional questionnaires were used alongside the QLQ-MY20, however, five were used between 2016 and 2020. No differences were observed in the types of treatments/therapies the QLQ-MY20 has been used to assess, the endpoint hierarchy that the QLQ-MY20 was selected for, the study phase it was used in or the presentation of QoL results in the form of tables, figures and/or in text.

The review of interventional study papers highlighted the main limitations with the PRO instruments or analysis/results as reported by authors (Table 2). Some issues are those generally affecting PROs rather than specific to the QLQ-MY20 such as differential dropout or poor completion rates potentially biasing the analysis, low baseline levels of symptoms limiting the opportunity to show improvement, single arm studies, short term PRO data collection and lack of standardization in collection and analysis of PROs across trials limiting comparison of results across studies. Issues raised which may be more specific to the QLQ-MY20 were the need for thresholds for meaningful change at the individual patient level, the need for consistency across studies in definitions of meaningful change, discrepancy between patient-reported ‘tingling hands and feet’ and the clinician reported peripheral neuropathy events, higher incidence of AEs or more severe AEs not translating into an impact on the PRO scores and potential lack of sensitivity of current questions to pick up variations in HRQoL depending on treatment administered. Another paper suggested that elements such as dosing convenience were currently not adequately measured by the available PROs.

### Role of QLQ-MY20 alongside clinical endpoints in RCTs

Table 3 summarises the results from the 15 RCTs with respect to comparisons of QLQ-MY20 scores between treatment groups. The statistical significance of any mean difference comparisons between groups and any time to deterioration (TTD) comparisons between groups is reported.

**Table 3:** Summary of QLQ-QLQ-MY20 results from 15 RCTs.

Study (Year)	RCT name and treatment comparison	N	QLQ-MY20 Subscale	Statistically significant mean difference?	Statistically significant TTD differences?	Evaluation of PRO results alongside clinical results in Abstract/Conclusion/Discussion	Further analyses of the QLQ-MY20 data
Verelst S. et al. [33]	HOVON49 MPT vs MP	284	DS	X	NA	“In conclusion, this prospective study shows that the higher frequency of adverse effects associated with MPT does not translate into a negative effect on the HRQoL as reported by patients and that MPT holds a better patient perspective. This can be interpreted as a favorable result because the current standard treatment of MPT for elderly patients with myeloma is not only known to have an improved clinical outcome but also no reduction in HRQoL.”	Association of clinical response and toxicity with QLQ-C30 and MY24 scores.
			SE	X	NA		
			FP	X	NA		
Dimopoulos M.A. et al. [46]*	Vantage 088 VOR + BTZ vs placebo	–	–	–	–	–	–
Dimopoulos M.A. et al. [34]	MM-015 MP vs MPR vs MPR-R (MP vs MPR-R primary HRQoL aim)	459	DS	X (Statistically significant symptom relief in both arms)	NA	“evidence of a favorable effect of MPR-R over MP in terms of Physical Functioning, and a clear trend in improvements in all other HRQoL domains tested, including Global QoL, Fatigue, Pain, and Disease Symptoms. The statistically non-significant change in Side Effects of Treatment scores from baseline in the MPR-R group and its comparability with scores in the MP group highlights the favorable tolerability profile of the MPR-R regimen, both during induction and maintenance.”	–
			SE	X	NA		
Weisel K. et al. [37]	MM-003 POM + LoDEX vs HiDEX	433	DS	NA	X	“These results report a real patient benefit: HRQoL is improved while patients receive therapy, and deteriorations in HRQoL are delayed in patients receiving POM þ LoDEX versus HiDEX.”	Proportion of patients improved/stable/worsened. Baseline scores versus best score prior to progression vs scores at progression.
			SE	NA	✓		
Delforge M. et al. [36]	FIRST Rd vs MPT	1623	DS	X (Statistically significant symptom relief in both arms)	NA	“Continuous lenalidomide and low-dose dexamethasone delays disease progression versus melphalan, prednisone, thalidomide and has been associated with a clinically meaningful improvement in health-related quality-of-life. These results further establish continuous lenalidomide and low-dose dexamethasone as a new standard of care for initial therapy of myeloma by demonstrating superior health-related quality-of-life during treatment, compared with melphalan, prednisone, halidomide.”	Effect of age on treatment group differences with respect to QLQ-C30 and QLQ-MY20 scores. Deterioration of HRQoL with progression.
			SE	✓	NA		
Stewart A.K. et al. [38]	ASPIRE KRd vs Rd	792	DS	x	x	“Results from the ASPIRE study confirm that the clinical benefits of the KRd triplet regimen, compared with the Rd doublet regimen, are associated with significant improvements in GHS/QoL, and there was no evidence of a detrimental impact from the triplet regimen on other aspects of HR-QoL.”	Proportion of patients improved/stable/worsened HRQoL by clinical response
			SE	x	x		
Ludwig H. et al. [48]	ENDEAVOR Kd vs Vd	929	DS	x	x	“The delay in time to deterioration was significantly longer for Kd56 versus Vd for global HR-QoL, physical, nausea/vomiting, and side effects. The doubling of progression-free survival in the ENDEAVOR trial is associated with a prolonged period of time before deterioration of HR-QoL in the Kd56 versus Vd group; this is particularly relevant given that patients’ HR-QoL steadily degrades as the disease progresses and patients relapse and develop resistance to therapy”	Proportion improved
			SE	✓	✓		
Richardson P. G. et al. 40]	PANORAMA-1 PAN + BTZ + DEX vs PBO + BTZ + DEX	147	DS	NA	NA	“The EORTC QLQ Myeloma module (EORTC QLQ-MY20) demonstrated initial improvements and subsequent stabilization of disease symptom scores in both arms and initial worsening and subsequent improvement of side effects of treatment scores, with the initial worsening more pronounced and recovery less pronounced with PAN + BTZ + DEX. Overall, these PRO findings support the addition of PAN to the BTZ + DEX regimen as an efficacious treatment option, with limited symptomatology and impact on patients’ QoL.”	Descriptive analysis only
			SE	NA	NA		
			FP	NA	NA		
			BI	NA	NA		
Royle K. L. et al. [41]	Myeloma IX CVAD vs CTD + ASCT (intensive) Or CTD vs MP (non-intensive)	1819	DS	NA	NA	“The results of this study showed that improvements in clinical outcomes were not at the detriment of patient reported HR-QoL. The findings are reassuring in the context of continuing development of sequential treatment for induction, consolidation and maintenance. However, such large-scale studies, this being the largest to date, are a major undertaking and very unlikely to be given priority in future studies. Indeed, it could be that more sensitive QoL instruments, or potentially instruments focussed on specific domains, for example neurological, will be required to identify clinically relevant differences between treatment combinations.”	–
			SE	NA	NA		
			FP	NA	NA		
			BI	NA	NA		
Cella D. et al. [42]	ELOQUENT-2 Eld vs Ld	646	DS	x	NA	“These findings show that previously reported improvements in progression-free survival and response rate with elotuzumab are achieved without detriment to HRQoL, which is maintained over time…Treatment responders showed more HRQoL and pain benefit than non-responders, supporting the clinical relevance of PROs in MM care.”	-
			SE	x	NA		
Leleu X. et al. [39]	TOURMALINE-MM1 IRd vs placebo-Rd	772	DS	X (Reduced in both arms)	NA	“Findings from this double-blind study demonstrate that addition of ixazomib to Rd significantly improved efficacy while HRQoL was maintained, reflecting the limited additional toxicity seen with IRd versus placebo-Rd, and support the feasibility of long-term IRd administration.”	Proportion of patients improved/stable/worsened Changes in HRQol by depth of response
			SE	X (Increased in both arms)	NA		
			FP	✓ (Increased in both arms, statistically significant greater improvements in IRd vs placebo-Rd at later timepoints)	NA		
			BI	x	NA		
Nielsen L. K. et al. [24]	Danish Myeloma Study Group CLAIM study Clarthromycin + VCD vs placebo + VCD	55	DS	x (Clinically relevant differences)	NA	“adding clarithromycin to the VCD regimen in patients with myeloma resulted in impaired HRQoL during the VCD induction phase continuing up to two months after HDT… The PRO data in the CLAIM study played a key role in explaining the causality link between the observed complications and the possible interaction between clarithromycin and bortezomib”	–
			SE	x (Clinically relevant differences)	NA		
			FP	x	NA		
			BI	✓ (Clinical and statistical relevant differences)	NA		
Ahmedzai S.H. et al. [43]	Myeloma X sASCT vs NTC	171	DS	x	NA	“The small and diminishing differences in Global health status and Side effects of treatment need to be considered alongside the results of Myeloma X, which showed a significant benefit of sASCT on OS. The benefits of sASCT should be considered alongside the relatively short-term negative effects on QoL and pain when making patient treatment decisions and further support the use of sASCT.”	Association between baseline scales and time to progression clinical outcome
			SE	✓	NA		
			FP	x	NA		
			BI	x	NA		
Moreau P. et al. [44]	ARROW Once-weekly Kd70mg/m2 vs twice-weekly Kd27mg/m2	469	DS	x	✓	“Collectively, the primary A.R.R.O.W. safety and efficacy data and the current PRO analysis reinforce that once-weekly Kd70 mg/m2 dose is superior and convenient while delivering more favorable HRQOL than the commonly used Kd27 mg/m2 dose. Thus, once-weekly Kd70 mg/m2 should be considered an important alternative to twice-weekly Kd27 mg/m2 in clinical practice.”	Proportion of patients improved/stable/worsened
			SE	x	x		
			FP	x	x		
Nielsen L.K. et al.	HOVON-87/NMSG18 MPT-T or MPR-R	596	DS	x	NA	“treatment with MPT-T and MPR-R improved HRQoL in elderly patients with NDMM and in general is clinically meaningful to the patients during maintenance therapy only. This supports the current paradigm of continuous treatment, not only improving survival, but also maintaining, and even improving, specific subscales of HRQoL.”	Proportion of patients improved/deteriorated Peripheral neuropathy analysed at the item level and showed statistically significant worsening during maintenance. All MY20 subscales except body image showed statistically significant changes over time within arms.
			SE	✓	NA		
			FP	x	NA		
			BI	✓ (maintenance only)	NA		

*ASCT* autologous stem cell transplant, *BTZ* bortezomib; *CTD* cyclophosphamide, thalidomide and dexamethasone; *CVAD* cyclophosphamide, vincristine, doxorubicin and dexamethasone; *DEX* dexamethasone; *ELd* elotuzumab, lenalidomide, and dexamethasone; *HiDEX* high-dose dexamethasone; *IRd* (ixazomib, lenalidomide (Revlimid) and dexamethasone); *Kd* (carfilzomib, dexamethasone); *KRd* (carfilzomib, lenalidomide (Revlimid) and dexamethasone); *Ld* (lenalidomide, and dexamethasone); *MP* (melphalan, Prednisone); *MPR* (melphalan, prednisone, lenalidomide); *MPR-R* (melphalan, prednisone, lenalidomide followed by lenalidomide maintenance); *MPT* (melphalan, prednisone, thalidomide); *MPT-T* (melphalan, prednisone, thalidomide followed by thalidomide maintenance); NA (not analysed); *NTC* (nontransplantation consolidation); PAN (panobinostat); *PBO* (placebo); *POM* + *LoDEX* (pomalidomide in combination with low-dose dexamethasone); *Rd* (lenalidomide, Revlimid, dexamethasone); *sASCT* (salvage autologous stem-cell transplantation); *VCD* (cyclophosphamide, bortezomib, dexamethasone); *Vd* (bortezomib, dexamethasone); *VOR* *+* *BTZ* (vorinostat, bortezomib); *X* indicates a statistically non-significant result; ✓ indicates a statistically significant result; NA indicates not tested or not reported in that trial; *Abstract only

Most trials evaluated the meaning of the PRO results in context with the clinical results. Five of the 15 trials were comparing triplet versus doublet therapy combination therapies. It was common in these studies for no statistically significant differences between treatment groups to be observed and for authors to interpret this as a positive result, demonstrating the addition of an agent to the combination did not impact on HRQoL. Four studies reported statistically significant differences between groups for the SE subscale (lenalidomide (Revlimid), dexamethasone [Rd] vs melphalan, prednisone, thalidomide [MPT], carfilzomib, dexamethasone [Kd] vs bortezomib, dexamethasone [Vd], melphalan, prednisone, thalidomide followed by thalidomide maintenance [MPT-T] vs melphalan, prednisone, lenalidomide followed by lenalidomide maintenance [MPR-R] and salvage autologous stem-cell transplantation [sASCT] vs nontransplantation consolidation [NTC]). One study reported longer time to deterioration for one arm for the DS subscale (once weekly vs twice weekly). One study reported longer time to deterioration for the SE subscale (Kd vs Vd). Another study reported differences between arms with respect to FP at later timepoints (IRd vs Rd). One small study [24] noted clinically relevant differences between cyclophosphamide‐bortezomib‐dexamethasone (VCD) plus placebo and VCD plus clarithromycin for DS and SE, and statistically significant differences with respect to BI.

In addition to these formal comparisons between treatment groups the RCTs also reported the proportion of patients with improved/stable/worsened QLQ-MY20 scores, association of clinical endpoints (response, time to progression and toxicity) with the QLQ-MY20 scales and the effect of age on HRQoL benefit.

### QLQ-MY20 validation studies

Nine validation studies were identified in the review [10, 17, 25–31]. Four validation studies highlighted potential ceiling effects for the BI subscale. No issues with item reliability (Cronbach’s alpha) were identified for the multi-item scales [10, 17, 25–27, 29, 30]. Test-retest reliability was assessed in one article [25]; all four QLQ-MY20 subscales had high test-retest reliability (ICC ≥ = 0.85). Two articles assessing factor analysis were inconsistent with one showing acceptable fit [25] and one suggesting item reduction in the SE subscale [27].

External validity convergent/discriminant validity was reported in two full text articles. Kontodimopoulos N. et al. (2012) demonstrated correlations between SF-36 domains and QLQ-MY20 domains, Graca Pereira M et al. (2019) found correlations between QLQ-MY20 domains and QlQ-C30 total score, Satisfaction with social support scale (SSSS), and the HADS.

Eight articles reported known groups validity across a range of groups (albumin, haemoglobin, beta 2 microglobulin [10, 17, 25–28, 30, 31], performance status, gender, age and presence of fractures). Three articles demonstrated ability to detect change [10, 25, 26].

## Discussion

The objective of this literature review was to review the use of the QLQ-MY20, since its first release 25 years ago, as the first validated module for patients with myeloma designed to be used with the EORTC QLQ-C30. The MM specific PRO measure consists of 20 items across four domains (refined from the original 24-item module [MY24] following early phase research). This literature review focused on the period after its publication in 1996 through to 2020.

There were a few drivers for this review. At the time of the original validation study the majority of clinical trials were in newly diagnosed patients and there was limited data for validation of the QLQ-MY20 in relapsed/refractory patients. Over the time period since the original publication of the QLQ-MY20, the treatment landscape has changed dramatically and patients with myeloma now undergo multiple lines of treatment and relapses. We wanted to use this review to see if the use of the questionnaire in relapsed patients has increased accordingly. The review aimed to summarise the range of studies the questionnaire has been reported in, how the data from the QLQ-MY20 was reported and how the results impacted on the evaluation of the treatments in the studies alongside clinical endpoints. We also wanted to collate any further psychometric evaluations of the QLQ-MY20 to see if any issues have emerged as the use of the questionnaire changed.

Seventy-four studies, that used the QLQ-MY20, were reviewed following screening, of which there were 15 RCTs, 6 single arm or cross-over trials, 44 observational and nine instrument validation studies, indicating diverse and extensive use of the QLQ-MY20 in several different clinical settings and investigations. The review of the published literature did not highlight any specific problems with the QLQ-MY20, however, qualitative interviews are ongoing to further explore the patient experience of symptoms and side effects of novel treatments. A revised version of the QLQ-MY20 is therefore warranted to ensure all concepts of interest are captured; concepts assessed by the additional COAs reported should be explored further in Phase I and II (generation of QOL issues and construction of the item list) of modular development and considered for inclusion in the updated version of the QLQ-MY20.

The RCTs highlighted that often no difference between treatments were observed with respect to the QLQ-MY20 subscales but that in conclusion often this was a desirable outcome, especially regarding the SE subscale (e.g., demonstrating that adding a further agent to a combination regimen does not have a detrimental impact on QoL). As new treatment regimens and new combination therapies continue to be developed, this should be a key consideration at the design stage for a RCT. The QoL comparisons should be non-inferiority rather than superiority and ensuring there is sufficient sample size to declare non-inferiority where applicable. It is also important for robust meaningful change thresholds to be determined in order that non-inferiority margins can be defined. To date there has been one study on deriving meaningful change [31] but further development of these may be required. The RCT data also supported the QLQ-MY20 subscales being related to clinical outcomes and supporting and supplementing the conclusions from the clinical endpoints. A number of studies investigated the relationship of the QLQ-MY20 scales with clinical outcomes such as time to progression and response.

Indicative of the expansion of the treatment portfolio and changing prognosis for patients, the proportion of RCTs using the QLQ-MY20 increased over time from n = 0 in the first 5 years to n = 10/13 in the last 5 years. The proportion of trials in patient post their 1st or subsequent relapses, relative to being newly diagnosed, increased over time from n = 1 in the first 5 years to n = 9/13 in the last 5 years. Over these time periods there were no observed trends for QoL endpoints to move up the hierarchy, however, this could be due to the inevitable time lag between research and publication of findings. Similarly, there were no trends or improvements in the reporting of QLQ-MY20 results in tables/figures rather than text alone; generally the reporting of the QLQ-MY20 included tables and/or figures throughout the period.

There were a few instances where limitations of the QLQ-MY20 were highlighted by individual papers. One issue was the need for work on meaningful change thresholds for the QLQ-MY20. Although this has since been addressed by Sully et al [31] more studies in this area would be beneficial in the future. Some studies used an additional peripheral neuropathy questionnaire alongside the QLQ-MY20 and one noted a discrepancy between the QLQ-MY20 item ‘tingling hands and feet’ and the clinician-reported peripheral neuropathy, which could indicate the need for more detailed items in the QLQ-MY20 on this side effect. Amongst the psychometrics studies, the instrument performed consistently well. One potential issue found in some studies was a ceiling affect for the BI subscale so this may warrant further investigation and may be the case for certain populations.

Potential limitations of our study include comprehensiveness of the usage of the QLQ-MY20. Our search will have identified any studies reporting results from the QLQ-MY20 but we acknowledge that this will exclude any studies that have used the instrument but not published any results from it. There will also be key multiple myeloma trials not in this review as they used only the QLQ-C30 or a different PRO. Regardless we have shown across a broad range of studies where the QLQ-MY20 has been used some of the trends over time in terms of patient populations and study designs.

In conclusion, the QLQ-MY20 has been shown to perform well psychometrically since its initial validation. The QLQ-MY20 scales have been supportive of clinical endpoints in RCTs and have been used to understand the patients’ QoL alongside improved response and time to progression outcomes. To maintain content validity in today’s MM treatment landscape (i.e., to ensure the instrument is relevant to MM patients and captures their symptoms and side effects of novel treatments and later lines of therapy) qualitative interviews with patients and health care professionals and an update to the QLQ-MY20 is underway to incorporate findings.

## Supplementary information

Supplementary Material

## Data Availability

All data generated or analysed during this study are included in this published article [and its supplementary information files].
