# Supplementary material for: Use of the European Organisation for Research and Treatment of Cancer multiple myeloma module (EORTC QLQ-MY20): a review of the literature 25 years after development
Source: Blood Cancer J. 2023 May 16;13(1):79. doi: 10.1038/s41408-023-00815-9 (PMC10188493; doi:10.1038/s41408-023-00815-9)
Supplement: Supplementary file 1 — Supplementary Material [file 41408_2023_815_MOESM1_ESM.docx]

**Supplementary material**

| Supplementary Table 1. Clinical outcome assessments used alongside the QLQ-MY20 in interventional and observational studies | | | |
| --- | --- | --- | --- |
| **Clinical outcome assessments** | **Total N=65** | **N=number of interventional studies** | **N=number of observational studies** |
| EQ-5D-5L | 6 | 3 | 3 |
| FACT-GOG-Ntx | 5 | 5 | 0 |
| BPI-SF | 5 | 1 | 4 |
| EORTC QLQ-CIPN20 | 5 | 0 | 5 |
| HADS | 4 | 0 | 4 |
| LANSS | 2 | 1 | 1 |
| EORTC IN-PATSAT32 | 2 | 0 | 2 |
| KPS | 2 | 0 | 2 |
| TSQM-9 | 1 | 1 | 0 |
| FACIT | 1 | 1 | 0 |
| EORTC QLQ-INFO25 | 1 | 0 | 1 |
| Mini-BESTest | 1 | 0 | 1 |
| FFS | 1 | 0 | 1 |
| BDI | 1 | 0 | 1 |
| EORTC QLQ-ELD15 | 1 | 0 | 1 |
| PHQ4 | 1 | 0 | 1 |
| EORTC QLQ-CIPN21 | 1 | 0 | 1 |
| SDS | 1 | 0 | 1 |
| ESAS-r | 1 | 0 | 1 |
| SF-SUNS | 1 | 0 | 1 |
| SF-12 | 1 | 0 | 1 |
| PSQI | 1 | 0 | 1 |
| GDS | 1 | 0 | 1 |
| Abbreviations: BDI (Beck Depression Inventory); BPI-SF (Brief pain inventory short form); EORTC (European Organisation for Research and Treatment of Cancer); EQ-5D-5L (EuroQOL-5 dimentions-5 Levels); ESAS-r (Edmonton Symptom Assessment System-revised); FACIT (The Functional Assessment of Chronic Illness Therapy); FACT-GOG-Ntx (Functional Assessment of Cancer Therapy/Gynecologic Oncology Group-Neurotoxicity); FFS (Fatigue Severity Scale); GDS (geriatric depression scale); HADS (Hospital Anxiety Depression Score); IN-PATSAT32 (cancer in-patient satisfaction with care 32 items); KPS (Karnofsky performance scale); Mini-BESTest (Balance Evaluation Systems Test); PHQ4 (Patient Health Questionnaire); PSQI (Pittsburgh sleep quality index); Quality of life questionnaire module for older people with cancer (EORTC QLQ-ELD15); QLQ-CIPN20 (quality of life questionnaire to assess chemotherapy-induced peripheral neuropathy twenty item scale); QLQ-CIPN21 (quality of life questionnaire to assess chemotherapy-induced peripheral neuropathy twenty-one item scale); QLQ-INFO25 (an instrument to assess the information given to cancer patients); SDS (Zung Self-Rating Depression Scale); SF-12 (12-Item Short Form Survey); SF-SUNS (Short-Form Survivor Unmet Needs Survey); S-LANSS (Leeds assessment of neuopathic symptoms and signs); TSQM-9 (Treatment Satisfaction Questionnaire for Medication) | | | |

| **Supplementary Table 2. Summary of the n=44 observational trials (cross-sectional or longitudinal/cohort) identified by the literature review. For each study identified, the summary table details the type of publication, author and year of publication, article/abstract title, study/design type, location of the study, the types of patients the QLQ-MY20 was used in, and additional questionnaires reported. The table is presented in chronology according to the paper’s year of publication.** | | | | | |
| --- | --- | --- | --- | --- | --- |
| **Year of publication** | **Title** | **Study design/type** | **Location of study author(s)** | **Patients newly diagnosed/ relapsing** | **Additional questionnaires reported** |
| **Publication type: Article** | | | | | |
| **Khalafallah A et al. 2011 (46)** | Quality of life assessment in multiple myeloma patients undergoing dose-reduced tandem autologous stem cell transplantation. | Longitudinal | Australia | Not reported | No additional questionnaires reported |
| **Tamburrelli F. et al. 2012 (47)** | Biphosphonate therapy in multiple myeloma in preventing vertebral collapse. preliminary report. | Cross-sectional | Italy | Not reported | No additional questionnaires reported |
| **Mols F. et al. 2012 (48)** | Health-related quality of life and disease-specific complaints among multiple myeloma patients up to 10 yr after diagnosis: Results from a population-based study using the PROFILES registry. | Longitudinal | Netherlands | Not reported | No additional questionnaires reported |
| **Acaster S. et al. 2013 (49)** | Impact of the treatment-free interval on health-related quality of life in patients with multiple myeloma: A UK cross-sectional survey. | Cross-sectional | UK | Newly diagnosed and 1st or subsequent relapses | EQ-5D-5L |
| **Jordan K. et al. 2014 (50)** | Effect of general symptom level, specific adverse events, treatment patterns, and patient characteristics on health-related quality of life in patients with multiple myeloma: Results of a European, multicenter cohort study. | Longitudinal | International | Newly diagnosed and 1st or subsequent relapses | No additional questionnaires reported |
| **Van der Poel M.W.M. et al. 2015 (51)** | Elderly multiple myeloma patients experience less deterioration in health-related quality of life than younger patients compared to a normative population: a study from the population-based PROFILES registry. | Cross-sectional | Netherlands | Not reported | No additional questionnaires reported |
| **Beijers A. et al. 2016**  **Dec 16 (52)** | Chemotherapy-induced peripheral neuropathy in multiple myeloma patients: Influence on quality of life and validation of a questionnaire for daily clinical practice. | Cross-sectional | Netherlands | Not reported | EORTC QLQ-CIPN20 |
| **Kiely F. et al. 2017 (53)** | Self-Reported Quality of Life and Symptom Burden in Ambulatory Patients With Multiple Myeloma on Disease-Modifying Treatment. | Cross-sectional | Ireland | Not reported | HADS |
| **Efficace F. et al. 2018 (54)** | A prospective observational study to assess clinical decision-making, prognosis, quality of life and satisfaction with care in patients with relapsed/refractory multiple myeloma: The CLARITY study protocol. | Longitudinal | Italy | 1st or subsequent relapses | EORTC QLQ-INFO25 |
| **Despiegel N. et al. 2019 (55)** | Health-Related Quality of Life of Patients With Multiple Myeloma Treated in Routine Clinical Practice in France. | Cross-sectional | France | Newly diagnosed and 1st or subsequent relapses | No additional questionnaires reported |
| **Balderas-Pena L.-M.-A. et al. 2019 (56)** | Health-Related Quality of Life and Satisfaction With Health Care: Relation to Clinical Stage in Mexican Patients With Multiple Myeloma. | Cross-sectional | Mexico | Newly diagnosed and 1st or subsequent relapses | EORTC IN-PATSAT32 |
| **Tay J. et al. 2019 (57)** | Health related quality of life for multiple myeloma patients according to treatment strategy after autologous stem cell transplant: a cross-sectional study using EORTC, EQ-5D and MY-20 scales. | Cross-sectional | Canada  USA | Not reported | EQ-5D-5L |
| **Servadio M. et al. 2020 (58)** | Physical activity and health-related quality of life in multiple myeloma survivors: the PROFILES registry. | Cross-sectional | Netherlands | Not reported | No additional questionnaires reported |
| **Shin Hoo Kim. et al. 2020 (59)** | The importance of physical function in patients with multiple myeloma for improving quality of life. | Cross-sectional | Korea | Not reported | Mini-BESTest  FFS  BDI |
| **Publication type: Conference abstract** | | | | | |
| **Petrucci M.T. Et al. 2009 (60)** | Costs and quality of life of multiple myeloma (MM) in Italy: The co.mi.m. study. | Cross-sectional | Italy | Newly diagnosed and 1st or subsequent relapses | No additional questionnaires reported |
| **Jordan K. et al. 2010 (61)** | Determinants of global QOL and physical and social functionality in multiple myeloma. | Cross-sectional | UK  Germany | Newly diagnosed and 1st or subsequent relapses | No additional questionnaires reported |
| **Cachia E. et al. 2012 (62)** | A study of pain, peripheral neuropathy and psychosocial late effects in patients with intensively treated advanced multiple myeloma. | Cross-sectional | UK | 1st or subsequent relapses | S-LANSS |
| **Eeltink C. et al. 2013 (63)** | A web-based tool to monitor chemotherapy induced peripheral neuropathy and quality of life. | Cross-sectional | Netherlands | Not reported | EORTC QLQ-ELD15  HADS |
| **Kyriakou C. et al. 2013 (64)** | Exploration and quantification of the determinants of baseline health-related quality of life (HRQOL) for patients in relapsed/refractory multiple myeloma. | Longitudinal | International | 1st or subsequent relapses | EORTC QLQ-CIPN20 |
| **Pamuk G.E. et al. 2013 (65)** | Assessment of quality of life in Turkish multiple myeloma patients by using EORTCQLQ-C30 and EORTC-QLQ-MY20. | Cross-sectional | Turkey | Not reported | HADS |
| **Petrucci M.T. et al. 2013 (66)** | Continued treatment duration, drug dosing and health-related quality of life (HRQoL) of patients with relapsed/refractory multiple myeloma (RRMM) receiving 2nd and 3rd line treatments: Results from a european multicentre study. | Longitudinal | International | 1st or subsequent relapses | No additional questionnaires reported |
| **Pezzullo L. et al. 2013 (12)** | Induction therapy with continuous alternate-day low dose lenalidomide combined with low-dose prednisone in octogenarian multiple myeloma patients. | Longitudinal | Italy | Newly diagnosed | No additional questionnaires reported* |
| **Alegre A. et al. 2014 (67)** | Observational prospective registry for the assessment of the clinical impact of starting anti-myeloma treatment at biological relapse. | Longitudinal | Spain | 1st or subsequent relapses | No additional questionnaires reported |
| **Boeckler J. et al. 2014 (68)** | Evaluation of individual Quality of Life in patients diagnosed with Multiple Myeloma by using standardized questionnaires of the European Organization for Research and Treatment of Cancer. | Longitudinal | Germany | Newly diagnosed and 1st or subsequent relapses | PHQ4 |
| **Miranda-Ruvalcaba C. et al. 2014 (69)** | Health related quality of life (HRQoL) in multiple myeloma patients treated in a tertiary referral hospital. | Cross-sectional | Mexico | Not reported | EORTC IN-PATSAT32 |
| **Vande Broek I. et al. 2014 (70)** | Longitudinal comparison of patients' and physicians' perceptions of patients' health-related quality of life in relapsed/refractory multiple myeloma. | Longitudinal | International | 1st or subsequent relapses | No additional questionnaires reported |
| **Fiala M.A. et al. 2015 (71)** | The association of international staging system (ISS) stage with disease and symptom burden in patients with newly diagnosed multiple myeloma. | Longitudinal | USA | Newly diagnosed | No additional questionnaires reported |
| **Fiala M.A. et al. 2015 (72)** | Variations in multiple myeloma disease presentation by race. | Cross-sectional | USA | Not reported | No additional questionnaires reported |
| **Keller J. et al. 2015 (73)** | Presenting characteristics and symptom burden of newly diagnosed older multiple myeloma patients in the commpass study. | Cross-sectional | USA | Not reported | No additional questionnaires reported |
| **Kyriakou C. et al. 2015 (74)** | Health-related quality of life in lenalidomide and bortezomib treated patients with relapsed/refractory multiple myeloma. | Longitudinal | International | 1st or subsequent relapses | EORTC QLQ-CIPN20 |
| **Leleu X. et al. 2015 (75)** | Levels of discordance between patients' and physicians' perceptions of patients' Health-Related Quality of Life (HRQoL) in relapsed/refractory multiple myeloma (RRMM)-a cross-cultural perspective. | Cross-sectional | International | 1st or subsequent relapses | EORTC QLQ-CIPN20 |
| **Petrucci M.T. et al. 2015 (76)** | Levels of discordance between patients' and physicians' perceptions of patients' health-related quality of life in relapsed/refractory multiple myeloma-a cross-cultural perspective. | Cross-sectional | International | 1st or subsequent relapses | EORTC QLQ-CIPN21 |
| **Rudzianskiene M. et al. 2015 (77)** | The impact of palliative radiotherapy on quality of life in multiple myeloma patients with painful bone destructions. | Longitudinal | Lithuania | Not reported | KPS |
| **Despiegel N. et al. 2016 (78)** | Quality of life of patients treated for multiple myeloma (MM) in France in a real-world setting. | Cross-sectional | France | Not reported | EQ-5D-5L |
| **Jo K. et al. 2016 (79)** | Quality of life in patients with multiple myeloma. | Cross-sectional | Korea | Not reported | No additional questionnaires reported |
| **Mess E. et al. 2016 (80)** | Grading quality of life in patients with multiple myeloma at the end of life. | Cross-sectional | Poland | Not reported | HADS  SDS  KPS  ESAS-r  BPI-SF |
| **Weger R. et al. 2016 (81)** | Routine assessment of patient-reported outcomes (PROs) in patients with multiple myeloma" An analysis of the Austrian Myeloma Registry (AMR). | Cross-sectional | Austria | Not reported | No additional questionnaires reported |
| **Faria S. et al. 2017 (11)** | Unmet needs and quality of life in Portuguese elderly myeloma patients. | Cross-sectional | Portugal | Not reported | SF-SUNS* |
| **Sidi Mohamed El Amine B. et al. 2017 (82)** | Quality of life and symptom burden in patients with multiple myeloma. | Cross-sectional | Algeria | Not reported | No additional questionnaires reported |
| **Nielsen L.K. et al. 2018 (83)** | Quality of life in newly diagnosed and relapsed Danish multiple myeloma patients-a national longitudinal study of Danish myeloma study group. | Longitudinal | Denmark | Newly diagnosed and 1st or subsequent relapses | EORTC QLQ-CIPN20  SF-12 |
| **Knauf W. et al. 2018 (84)** | MYRIAM: Prospective, intersectoral Real World cohort study for treatment and outcome of myeloma patients in Germany. | Longitudinal | Germany | Not reported | BPI-SF |
| **Tavitian E. et al. 2018 (85)** | Continuous mobile wearable bio-monitoring of newly diagnosed multiple myeloma patients undergoing initial chemotherapy. | Longitudinal | USA | Newly diagnosed | BPI-SF |
| **Kim S.J. et al. 2018 (86)** | The assessment of health-related quality of life in relapsed or refractory myeloma patients receiving lenalidomide and low dose dexamethasone: A result of hola study. | Longitudinal | Korea | 1st or subsequent relapses | PSQI  GDS  BPI-SF |
| *Publication did not report use of the EORTC QLQ-C30  Abbreviations: AMR (Austrian Myeloma Registry); BDI (Beck Depression Inventory); BPI-SF (Brief pain inventory short form); CCI (Charlson Comorbidity Index); EORTC (European Organisation for Research and Treatment of Cancer); EQ-5D-5L (EuroQOL-5 dimentions-5 Levels); ESAS-r (Edmonton Symptom Assessment System-revised); FFS (Fatigue Severity Scale); GDS (geriatric depression scale); HADS (Hospital Anxiety Depression Score); HRQOL (Health-related quality of life); IADL (Lawton Instrumental Activities of Daily Living); IN-PATSAT32 (cancer in-patient satisfaction with care 32 items); Katz ADL (Katz Index of Independence in Activities of Daily Living); KPS (Karnofsky performance scale); Mini-BESTest (Balance Evaluation Systems Test); MM (Multiple myeloma); PHQ4 (Patient Health Questionnaire); PSQI (Pittsburgh sleep quality index); Quality of life questionnaire module for older people with cancer (EORTC QLQ-ELD15); QLQ-CIPN20 (quality of life questionnaire to assess chemotherapy-induced peripheral neuropathy twenty item scale); QLQ-CIPN21 (quality of life questionnaire to assess chemotherapy-induced peripheral neuropathy twenty-one item scale); QLQ-INFO25 (an instrument to assess the information given to cancer patients); RRMM (Relapsed/refractory multiple myeloma); SDS (Zung Self-Rating Depression Scale); SF-12 (12-Item Short Form Survey); SF-SUNS (Short-Form Survivor Unmet Needs Survey); S-LANSS (Leeds assessment of neuopathic symptoms and signs); TSQM-9 (Treatment Satisfaction Questionnaire for Medication) | | | | | |
